# Haptoglobin Induces a Specific Proteomic Profile and a Mature-Associated Phenotype on Primary Human Monocyte-Derived Dendritic Cells

**DOI:** 10.3390/ijms23136882

**Published:** 2022-06-21

**Authors:** Alfredo Torres, Sheilah Vivanco, Francisca Lavín, Cristián Pereda, Alexey Chernobrovkin, Alejandra Gleisner, Marcela Alcota, Milton Larrondo, Mercedes N. López, Flavio Salazar-Onfray, Roman A. Zubarev, Fermín E. González

**Affiliations:** 1 Laboratory of Experimental Immunology & Cancer, Faculty of Dentistry, University of Chile, Santiago 8380492, Chile; atorres@odontologia.uchile.cl (A.T.); svivanco@ug.uchile.cl (S.V.); franlavin90@gmail.com (F.L.); 2 Department of Conservative Dentistry, Faculty of Dentistry, University of Chile, Santiago 8380492, Chile; malcota@u.uchile.cl; 3 Disciplinary Program of Immunology, Institute of Biomedical Sciences, Faculty of Medicine, University of Chile, Santiago 8380453, Chile; cperda@gmail.com (C.P.); alejandra.gleisner@gmail.com (A.G.); melopez@uchile.cl (M.N.L.); fsalazar@u.uchile.cl (F.S.-O.); 4 Department of Medical Biochemistry and Biophysics, Karolinska Institute, SE17177 Stockholm, Sweden; alexey.chernobrovkin@ki.se (A.C.); roman.zubarev@ki.se (R.A.Z.); 5 Blood Bank Service, University of Chile Clinical Hospital, Santiago 8380453, Chile; mlarrondo@hcuch.cl; 6 Millennium Institute on Immunology and Immunotherapy, Faculty of Medicine, University of Chile, Santiago 8380453, Chile

**Keywords:** haptoglobin, DAMPs, dendritic cells, melanoma cell lysates

## Abstract

Damage-associated molecular patterns (DAMPs) play a critical role in dendritic cells (DCs) ability to trigger a specific and efficient adaptive immune response for different physiological and pathological scenarios. We have previously identified constitutive DAMPs (HMGB1 and Calreticulin) as well as new putative inducible DAMPs such as Haptoglobin (HP), from a therapeutically used heat shock-conditioned melanoma cell lysate (called TRIMEL). Remarkably, HP was shown to be the most abundant protein in the proteomic profile of heat shock-conditioned TRIMEL samples. However, its relative contribution to the observed DCs phenotype has not been fully elucidated. Human DCs were generated from monocytes isolated from PBMC of melanoma patients and healthy donors. DC lineage was induced with rhIL-4 and rhGM-CSF. After additional stimulation with HP, the proteome of these HP-stimulated cells was characterized. In addition, DCs were phenotypically characterized by flow cytometry for canonical maturation markers and cytokine production. Finally, in vitro transmigration capacity was assessed using Transwell plates. Our results showed that the stimulation with HP was associated with the presence of exclusive and higher relative abundance of specific immune-; energy production-; lipid biosynthesis-; and DAMPs-related proteins. Importantly, HP stimulation enhanced the expression of specific DC maturation markers and pro-inflammatory and Th1-associated cytokines, and an in vitro transmigration of primary human DCs. Taken together, these data suggest that HP can be considered as a new inducible DAMP with an important role in in vitro DC activation for cancer immunotherapy.

## 1. Introduction

DCs are professional antigen-presenting cells (APCs) that can efficiently trigger an adaptive immunity against both pathogens and tumors [1,2,3,4]. This is broadly done by their ability to efficiently capture and process antigens (Ags), in order to respond upon sensing either pathogen- or self-derived danger signals in peripheral tissues, and to migrate to draining lymph nodes (LN), among others. Here, DCs can engage, in a proper co-stimulatory signal and cytokines context, Ag-specific naïve CD4^+^ and CD8^+^ T cells, forming an immunological synapse and, in turn, triggering their activation, proliferation, and homing to peripheral tissues to perform their effector functions [5,6,7,8]. In this scenario, DCs are considered as translators between innate and adaptive immune responses to adequately target different insults [9].

One of the most relevant DC abilities is their strong capacity to sense the milieu at the injury site, and to transduce this information to naïve T cells in order to trigger adaptive immunity [10]. This ability of DCs to transduce information is mainly achieved through a myriad of innate-related pattern recognition receptors (PRRs) such as Toll like receptors (TLRs), C-type lectin receptors (CLRs), Retinoic acid-inducible gene (RIG)-I-like receptors (RLRs), NOD-like receptors (NLRs), scavenger receptors, and phagocytosis receptors, among others [11,12,13], expressed by these cells. Upon engagement with their ligands, these receptors trigger several signaling pathways in immature DCs (iDCs), which, in turn, activate different abilities associated with a DC mature phenotype and with their capacity to induce a proper adaptive immune response to efficiently control the injury [11,12]. In this context, the functional maturation process is a critical feature of DC biology, which is characterized by the acquisition of relevant properties such as the downregulation of periphery homing and phagocytosis receptors [9], the upregulation of immunoproteasome activity [14,15], the migratory capacity [9,16,17], pro-inflammatory cytokine secretion, and Ag presentation-associated molecules [9,17,18], among others.

The recognition that DCs are important regulators of immune responses [9], together with the development of in vitro techniques to obtain large numbers of DCs from isolated monocytes [19], has stimulated research on DC-based vaccination strategies. DC-based vaccines are likely to be safe, relatively inexpensive, and, most importantly, provide long lasting protective immunity. During the last decade, we have developed a tumor Ag presenting cell (TAPCells)-based immunotherapy against malignant melanoma (MM). TAPCells are generated from autologous monocytes and use the heat shock-conditioned melanoma cell lysate TRIMEL, which can rapidly induce a mature and committed DC phenotype from cytokine-activated monocytes (AM) [18,20]. TAPCells induce T cell-mediated immune responses leading to improved long-term survival of about 60% of stage IV MM patients in studies involving more than 150 patients [5,18,21,22,23]. However, about 40% of the treated patients were non-responders, with a significantly lower survival rate compared with responders [18,21]. This constraint is likely due to the absence of sufficient amounts of immunogenic danger signals either during ex vivo DC generation or immunization, or deficiencies in Ag processing and presentation by injected DCs, which, at least in part, can also be due to deficient danger signal stimuli [18].

DAMPs are evolutionary conserved endogenous factors that are secreted, released, or surface exposed by dying, stressed, or injured cells that can act as “danger signals” changing the tissue microenvironment and inducing an inflammatory response [24,25]. DAMPs consist of a heterogeneous group of molecules with a distinct nature, which includes ATP, uric acid, heat shock proteins (HSPs), nucleic acids (DNA and RNA), HMGB1 and calcium-binding factors (proteins of the S100 family) [25], Amyloid b peptide [26] and F-Actin [27], among others. DAMPs are recognized by all immune cells types, most notably DCs, through PRRs on their surface (e.g., TLRs) [28] or in their cytoplasm (e.g., NLRs) [13]. A remarkable characteristic of DAMPs is that most of these molecules have completely distinct non-immunological related functions under normal physiological conditions [29] and, upon release, they change their functionality, being recognized by PRRs and activating immune cells. Importantly, recent findings have established that the specific DAMPs sensed by DCs at the periphery can be considered as signal 0 and, in addition, the specific death program from which these DAMPs were released (e.g., apoptosis vs. necroptosis) can be considered signal −1 for an adequate immunological synapse because they can dramatically change the phenotype and functional capacity of DCs to induce a proper CD4^+^ and CD8^+^ T cell activation and polarization [10]. 

We have previously demonstrated that DAMPs present in the lysate TRIMEL are likely responsible for an efficient Ag cross-presentation by ex vivo produced TAPCells [18]. Moreover, we have also reported that the HS conditioning of melanoma cells belonging to the lysate increased Calreticulin (CRT) plasma membrane translocation and induced the release of HMGB1. Both CRT and HMGB1 mobilization were associated with enhanced DCs’ maturation and an efficient Ag cross-presentation capacity, respectively [18]. Additionally, HMGB1 from the lysate TRIMEL co-localizes with monocyte TLR4, and the blockade of TLR4 inhibits the expression of maturation-associated markers, pro-inflammatory cytokines and the receptor C-C chemokine receptor type 7 (CCR7) induced by the lysate [22]. DCs’ ability to migrate to draining lymph nodes, a relevant pre-requisite for their clinical efficacy, is also increased upon HS-MCL stimulation [16]. Recently, we have demonstrated the effect of a TRIMEL-based vaccine (TRIMELVax) in promoting conventional DCs (cDCs) and inhibiting tumor growth in a preclinical model [30]. In this context, it is relevant to unravel the effect that specific DAMPs released from cancer cells induce in the maturation and function of DCs.

HP was previously identified as a putative DAMP protein from the therapeutically used lysate TRIMEL [31]. HP is a plasmatic glycoprotein of 38 kDa. Its main function is to bind hemoglobin (Hb), forming a stable complex HP-Hb, which is cleared via CD163-mediated endocytosis and thus preventing the oxidative tissue damage induced by free Hb [32,33], which has also been described with DAMP-associated roles [34]. Importantly, HP activation of DCs was shown in a murine model of skin transplantation [35]. Recently, an amplifying role of HP in inflammation after cardiac transplantation in a murine model has also been described, demonstrating an interaction between HP and the immune system [36]. Remarkably, HP was identified by our group as the most abundant protein in the proteomic profile of HS-MCL samples [31]. However, its relative contribution to the observed phenotype of the ex vivo generated DCs has not been fully elucidated. Here, the proteome of HP-stimulated DCs showed a higher relative abundance of specific proteins associated with the processes of antigen processing and presentation, lipid biosynthesis, and energy production. Moreover, there were proteins associated with DAMPs functions, cell shape, and migration. Finally, HP stimulation was associated with an enhanced expression of specific DCs maturation markers and the in vitro transmigration of primary human DCs, all features related to a mature DC (mDC) phenotype and functionality.

## 2. Results

### 2.1. Ex Vivo Generated Human Primary DCs Show a Specific Proteomic Profile under HP Stimulation

To analyze the effect of HP stimulation on primary human DC phenotype, we perform a high throughput proteomic study to AM and HP- or TRIMEL-stimulated cells.

**Figure 1:**
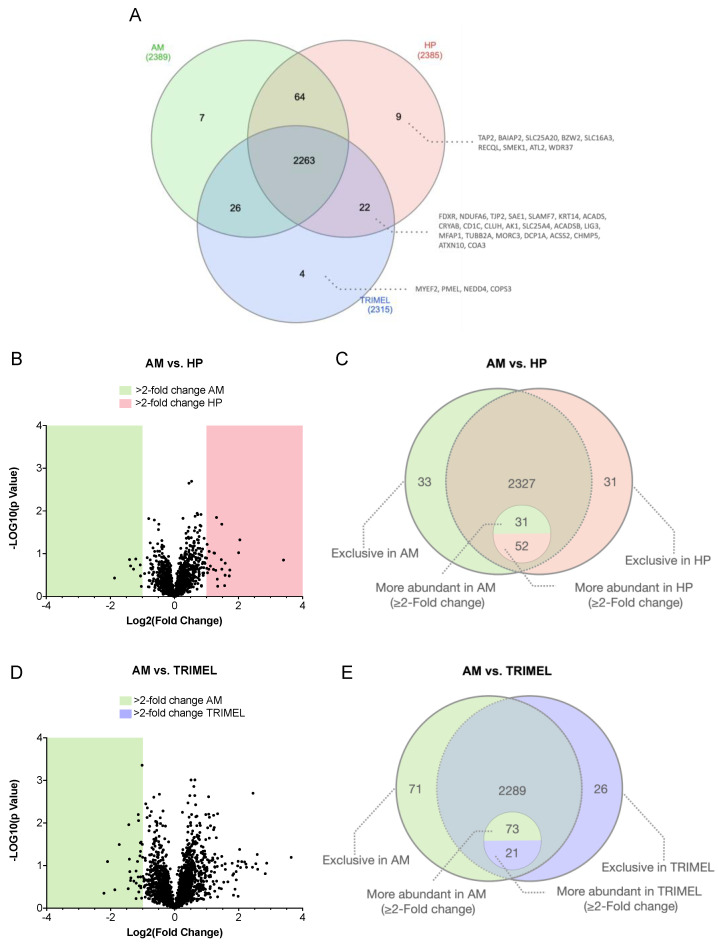
Qualitative and quantitative proteomic comparison of AM, HP-, and TRIMEL-stimulated cells. (**A**) The Venn diagram of the identified proteins shows that a total of 2395 proteins were identified. In addition, 2263 proteins were present in the three studied conditions. Furthermore, 22 proteins were simultaneously found in HP- and TRIMEL-stimulated cells; 64 in HP and AM; and 26 in TRIMEL and AM cells. Nine proteins were exclusively identified in HP-stimulated cells, four in TRIMEL-stimulated cells, and seven in AM cells. (**B**) Volcano Scatter Plot of the proteins with the highest abundance (≥2 fold-change) from AM vs. HP, and (**C**) AM vs. TRIMEL. The plots were constructed based on the variation in the fold change of each protein (Log2) and the *p*-value (−Log10) when comparing both groups. (**D**) Representation of the exclusive and more abundant (≥2 times) proteins between HP and AM cells. In addition, 2327 proteins were present in both groups. Of them, 52 proteins were more abundant in HP samples and 31 in AM and 31 proteins were only identified in HP and 33 in AM; (**E**) representation of the exclusive and more abundant proteins between TRIMEL and AM cells. A total of 2289 proteins were found in both groups. Twenty-one proteins were more abundant in TRIMEL samples and 73 in AM cells. Twenty-six proteins were exclusively identified in TRIMEL-stimulated cells and 71 in AM cells.

When considering the total of studied samples, a total amount of 2395 proteins were identified. Of them, 2263 were identified to be present in the three analyzed conditions (AM, TRIMEL- and HP-stimulated cells). The comparison among the three conditions showed that nine proteins were exclusively identified in HP-stimulated cells (TAP2, BAIAP2, SLC25A20, BZW2, SLC16A3, RECQL, SMEK1, ATL2, WDR37), four proteins in TRIMEL-stimulated cells (MYEF2, PMEL, NEDD4, COPS3), and seven in AM cells (KIAA1033, SEC61A1, MITF, TMED2, MAPRE2, CECR5, PEF1) (Figure 1A; Supplementary Table S1). Importantly, compared to AM cells, 22 proteins were identified to be exclusively induced by both HP and TRIMEL stimulations (FDXR, NDUFA6, TJP2, SAE1, SLAMF7, KRT14, ACADS, CRYAB, CD1C, CLUH, AK1, SLC25A4, ACADSB, LIG3, MFAP1, TUBB2A, MORC3, DCP1A, ACSS2, CHMP5, ATXN10, COA3) (Figure 1A and Supplementary Table S1). The abundance ratios between HP vs. AM, TRIMEL vs. AM and TRIMEL vs. HP are shown in Supplementary Table S2. Interestingly, the abundance ratios of different proteins vary among groups, probably because TRIMEL is a highly complex melanoma cell lysate with a great number of regulatory molecules which confers the lysate with its high stimulation capacity.

The comparison between AM and HP showed that 31 proteins were exclusively identified, and 52 proteins were more abundant in HP-stimulated cells (Figure 1B,C, Table 1 and Supplementary Table S3). Furthermore, 33 proteins were exclusive and 31 proteins were more abundant in AM cells (Figure 1B,C; Supplementary Table S4). When comparing AM vs. TRIMEL, we found 26 proteins exclusively identified in TRIMEL-stimulated cells and 21 more abundant proteins in the same group (Figure 1D,E; Supplementary Table S5).

**Figure 2:**
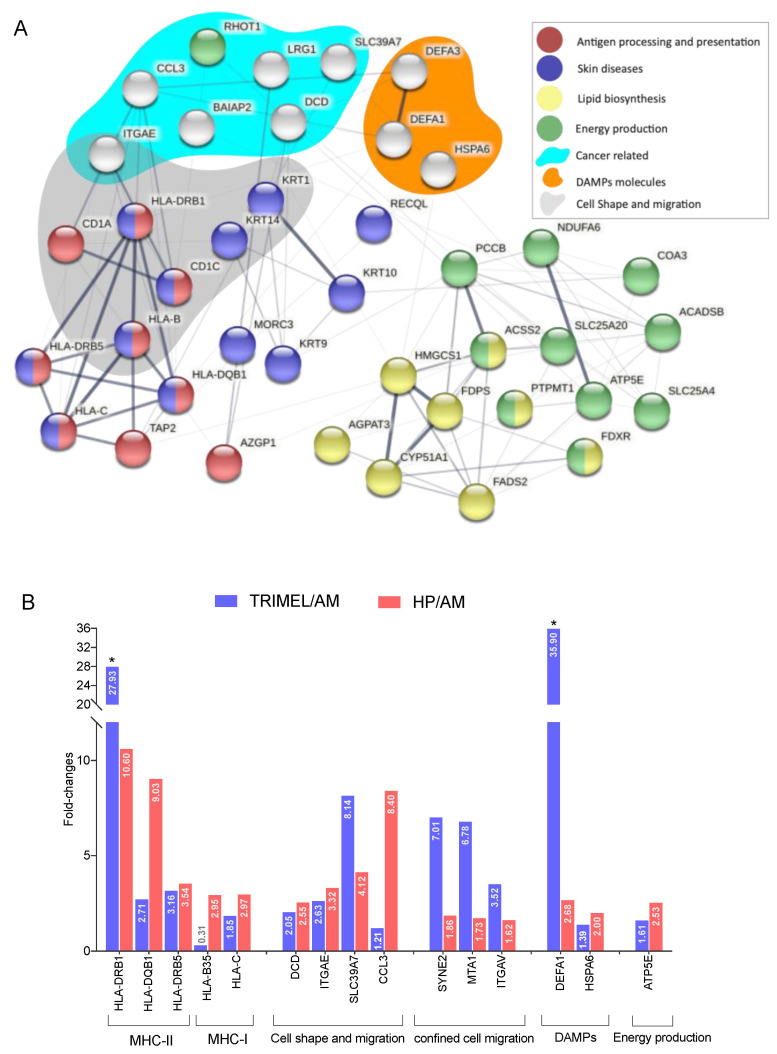
Protein–protein interaction network and fold change of relevant proteins in HP- and TRIMEL-stimulated cells. (**A**) Protein interactome of exclusive and more abundant proteins in HP-stimulated cells compared with AM. Interactome of proteins selected from a differentiated panel of 83 proteins, of which 31 are exclusive and 52 are in greater abundance (≥2 fold-change), shows proteins related to antigen processing and presentation (red), energy production (green) and lipid biosynthesis (yellow). In addition, proteins associated with cancer development (cyan shadow), changes in cell shape and cell migration (grey shadow), and proteins with DAMP functions (orange shadow) were also identified. Proteins were found more abundant considering a cutoff *p* < 0.01 or abs (log2 (FC)) > 1; (**B**) comparison of TRIMEL/AM and HP/AM fold-changes of selected proteins. In both TRIMEL- and HP-stimulated cells, there were several proteins with a higher abundance regarding AM condition. In the TRIMEL group, the most abundant proteins were DEFA1 (35.9 fold-change), HLA-DRV1 (27.93 fold-change) and SLC39A7 (8.14 fold-change), while, in the HP group, the most abundant proteins were: HLA-DRB1 (10.8 fold-change), HLA-DQB1 (9.03 fold-change) and CCL3 (8.4 fold-change). Confined cell migration related proteins were more abundant in the TRIMEL group compared with HP-stimulated cells. * *p* < 0.05.

The protein interaction network of more abundant and exclusive proteins from HP-stimulated cells showed the presence of proteins associated with the processes of antigen processing and presentation (HLA-DRB1, HLA-DQB1, HLA-DRB5, HLA-C, HLA-B, AZGP1, CD1A, CD1C, and TAP2), lipid biosynthesis (HMGCS1, PTPMT1, AGPAT3, FDPS, CYP51A1, FDPS, FDXR, and ACSS2) and energy production (PTPMT1, ACSS2, FDXR, PCCB, NDUFA6, SLC25A4, SLC25A20, ATP5E, ACADSB, and COA3). In addition, there were proteins associated with DAMPs functions (DEFA1, DEFA3, and HSPA6) and cell shape and migration (CD1A, CD1C, KRT1, KRT14, HLA-B, HLA-DRB1, and ITGAE), all features related to mature DC functionality (Figure 2A).

Interestingly, in some cases and compared with AM cells, the fold change of specific proteins induced by HP was higher than the one induced by TRIMEL (e.g., HLA-DQB1, HLA-B35, CCL3, and ATP5E) (Figure 2B).

### 2.2. Haptoglobin Enhances the In Vitro Expression of DCs Maturation Markers and the Secretion of Proinflammatory and Th1-Related Cytokines on Primary Human iDC

Previously, we have reported that the in vitro stimulation with the melanoma cell lysate TRIMEL to primary human AM cells mediated up to four-fold induction of several surface markers associated with DCs maturation such as MHC-I, MHC-II, CD80, CD83, and CD86 [18]. In addition, TRIMEL could also significantly induce a two-fold increase in the expression of MHC-II, CD83 and CCR7 molecules in monocyte/macrophage THP-1 cells, generating a DC-like phenotype when compared with the unstimulated control cells [16].

**Figure 3:**
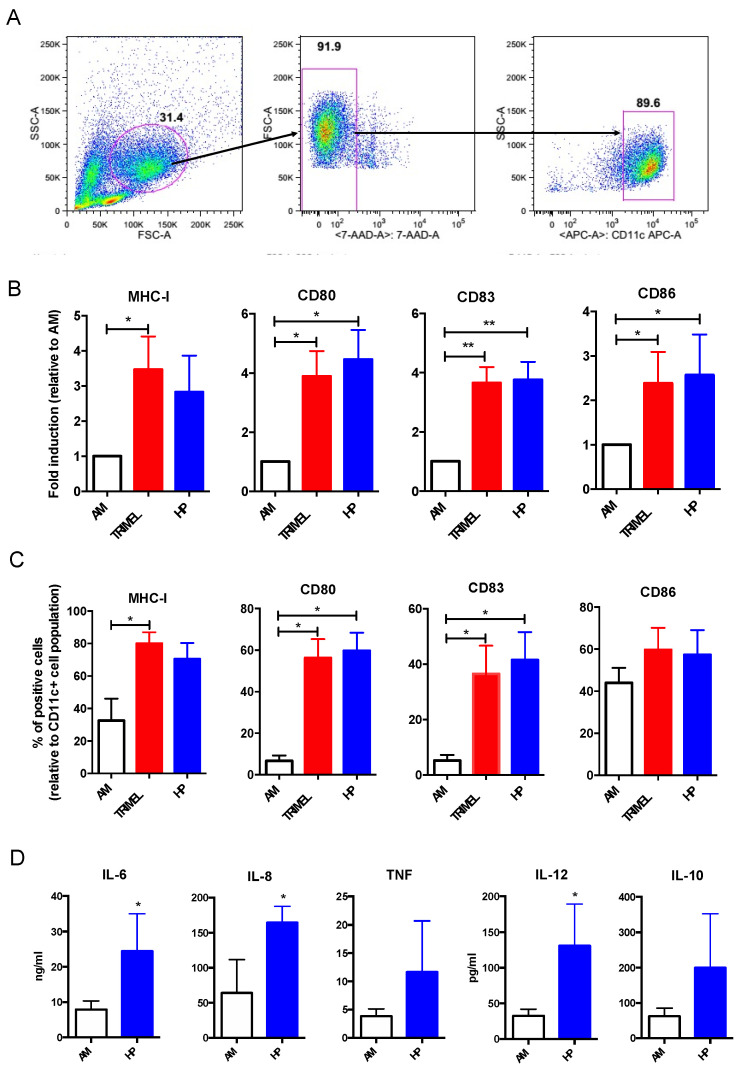
In vitro effects of HP on primary human monocyte-derived iDCs phenotype and cytokine production. DC lineage was induced with IL-4 (500 U/mL), and GM-CSF (800 U/mL) as previously described [18], before additional stimulation with HP and HS-MCL (TRIMEL) lysate as control. (**A**) gating strategy; (**B**) MHC-I, CD80, CD83 and CD86 expression in unstimulated cells (AM), TRIMEL-stimulated cells (50 μg/mL), and HP-stimulated cells (10 ng/mL); (**C**) percentage of MHC-I, CD80, CD83, and CD86 positive cells in AM, TRIMEL-stimulated cells (50 μg/mL), and HP-stimulated cells (0,1 and 10 ng/mL); (**D**) IL-12, TNF-α, IL-10, IL-6, and IL-8 secretion. The concentration of cytokines was analyzed in supernatants obtained from 24 h co-cultures of AM or HP-stimulated iDCs with CD-40L expressing fibroblast (1:1 ratio). * *p* < 0.05; ** *p* < 0.01.

To evaluate the capacity of HP to induce a mature DC phenotype, we stimulated primary human AM cells with TRIMEL and HP for 24 h. After a specific gating strategy (Figure 3A), the canonical DC maturation-associated markers MHC-I, CD80, CD83, and CD86 were evaluated. CD80, CD83, and CD86 maturation markers showed a higher expression in HP-stimulated iDCs compared with AM cells and an equivalent expression compared with TRIMEL-stimulated cells (Figure 3B). When comparing the percentage of positive cells, CD80 and CD86 positive cells were higher in HP-stimulated cells in comparison with control AM cells and equivalent to the percentage of positive cells in TRIMEL-stimulated cells (Figure 3C).

Proinflammatory and Th1-associated cytokine expression is a hallmark of conventional DCs [9,37]. In this context, we evaluated the cytokine expression induced by HP stimulation in human primary iDCs. To this, supernatants obtained from 24 h co-cultures of AM or HP-stimulated cells with CD-40L expressing fibroblast (1:1 ratio) were evaluated for IL-12, TNF-a, IL-10, IL-6, and IL-8 concentrations. HP induces a significantly higher expression of the Th1 profile-associated cytokine IL-12 (Figure 3D). Moreover, the expression of the proinflammatory cytokines IL-6 and IL-8 was also higher in HP-stimulated primary iDCs when compared with AM cells (Figure 3D).

### 2.3. HP Induces the Expression of the Lymph Node (LN) Homing Receptor CCR7 and the In Vitro Transmigration of Primary Human iDCs

**Figure 4:**
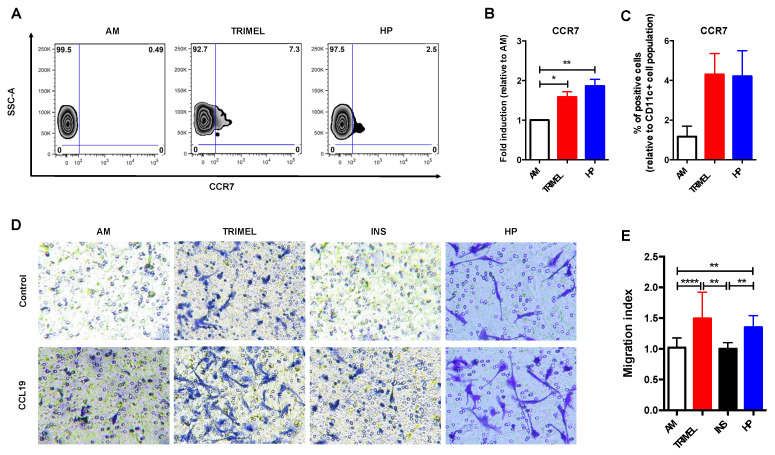
CCR7 expression and in vitro transmigration capability in HS-stimulated primary human iDCs. (**A**) Representative zebra plots showing the percentage of CCR7 positive cells in AM and HP-stimulated cells (1 and 10 ng/mL); (**B**) CCR7 expression in unstimulated cells (AM), TRIMEL-stimulated cells (50 μg/mL), and HP-stimulated cells (10 ng/mL); (**C**) percentage of CCR7 positive cells in AM, TRIMEL-stimulated cells (50 μg/mL), and HP-stimulated cells (0,1 and 10 ng/mL); (**D**) representative images from Transwell experiments of different conditions: AM, TRIMEL, INS, and HP in response to the canonical CCR7 ligand, the chemokine CCL19 (10 ng/mL) or AIM-V (negative control); (**E**) quantification of the in vitro transmigration of AM and stimulated cells with TRIMEL (50 μg/mL), Insulin (10 ng/mL), and HP (10 ng/mL). Data are expressed as a migration index, which is the ratio between the number of migrated cells towards CCL19 and the number of migrated cells towards the medium alone for each cell condition studied. * *p* < 0.05; ** *p* < 0.01; **** *p* < 0.0001.

We have previously demonstrated that TRIMEL can rapidly (~24 h) induce a mature and committed DC-phenotype in TAPCells and their in vitro and in vivo migration from the injection site to draining lymph nodes [16,18], a fundamental ability of DCs to trigger a proper adaptive immunity. In this scenario, we focused on establishing the role of HP in the in vitro migration capacity of human primary DCs. To this end, we evaluated the effect of HP on the induction of the chemokine receptor CCR7, another well-established maturation marker of DCs, essential for the migration of DCs from the periphery to draining lymphatic tissues. Our results showed that an important proportion of HP-stimulated iDCs, when compared to AM cells, acquired surface expression of CCR7 molecules (Figure 4A,C). Moreover, there was a significant 1,9-fold increase in the expression levels of CCR7 when the mean fluorescence intensity (MFI) was evaluated in HP-stimulated cells and AM derived from the monocytes of at least three different melanoma patients (Figure 4B).

Furthermore, we investigated the effect of HP stimulation in activated monocytes in response to the chemokine CCL19, one of the canonical CCR7 ligands. To this end, we used an in vitro transmigration assay through a polycarbonate membrane in a double well chamber (Figure 4D). We observed that HP-stimulated cells significantly increased their transmigration index in response to CCL19 when compared to AM and INS control cells (Figure 4E). Our data suggest that HP induces a mature DC-like phenotype characterized by an increased expression of the receptor CCR7, which in turn augmented human iDCs in vitro transmigratory potential in response to CCL19.

## 3. Discussion

Pluricellular organisms have developed complex systems to deal with cellular stress induced by pathogen invasion, cancer development, or injury. In this context, protein DAMPs are highly conserved proteins that are released during cellular stress to help in the initiation of an efficient immune response and reparative processes [38]. Indeed, stressed or dying cells release active or passively intrinsic DAMPs molecules, which are recognized by various PRRs, to elicit a controlled immune-inflammatory response resulting in the clearance of dysfunctional cell without compromising the survival of the host [25,31,39]. 

In order to establish some DAMP-associated properties of HP, the proteome of in vitro HP-stimulated primary human DCs was qualitatively and quantitatively evaluated. In addition, the in vitro expression of canonical mDCs-associated markers, cytokines production, and transmigration ability of these primary monocyte-derived DCs were also studied. 

Among the proteins induced in the proteome of in vitro generated DCs by the stimulation with HP, we found several proteins with previously described functions associated with antigen processing and presentation (CD1A, CD1C, HLA-DRB1, HLA-DRB5, HLA-DQB1, HLA-B, HLA-C, TAP2, and AZGP1). A higher expression of MHC class I and II molecules is a well-known hallmark of DC maturation [5,16,18,40]. In this context, the finding of several exclusive and more abundant proteins associated with antigen processing and presentation processes correlates with a more mature phenotype induced by HP. 

CD1a is a Langerhans cell (LC) marker, and HP has been described to prevent functional maturation of epidermal LCs in the skin [41]. Interestingly, and analogous to our model, the source of HP is skin keratinocytes, since LC are unable to synthesize the protein by themselves [42]. TAP2 protein, alongside with TAP1, is one of the heterodimeric subunits of the transporter associated with antigen processing (TAP) protein complex, which is a main part of the translocation machinery that transports peptide antigens generated by the immunoproteasome and suited for MHC-I loading from the cytosol to the ER lumen [43]. In this context, the TAP protein complex is fundamental for a proper cross-presentation, one of the most characteristic abilities of mature conventional DCs [44]. In addition, CD1C is a well-recognized conventional DC type 2 marker that is able to present antigens to both CD4^+^ and CD8^+^ T cells [45]. 

In recent years, the fundamental relevance of metabolic changes during the DC maturation process and functionality has been highlighted [46,47]. Interestingly, we found several energy production-associated proteins that were induced by HP-stimulation (RHOT1, PCCB, NDUFA6, COA3, ACADSB, SLC25A20, SLC25A4, ATP5E, ACSS2, PTPMT1, and FDXR). For instance, RHOT1 is involved in mitochondrial dynamics and homeostasis, and has also been involved in the control of migration and polarity of lymphocytes [48]. Indeed, RHOT1 has been associated with mitochondrial positioning at the leading edge providing localized energy that enhances cell migration [49], which is also a relevant feature of mature DCs. 

The protein NADH: ubiquinone oxidoreductase subunit A6 (NDUFA6), is a subunit of complex I, the largest mitochondrial respiratory chain complex, being essential for correct complex assembly [50]. Interestingly, it has been described that NDUFA6 participates in different processes of cell differentiation such as the regulation of the adipocyte lineage differentiation via the stearoyl-CoA desaturase 1 (Scd1) protein and in muscle cell differentiation as a target of the transcription factor TEAD1 [51,52]. Another interesting protein is the cytochrome c oxidase (COX) assembly factor COA3, which is involved in promoting a Warburg-like effect in cancer cells [53]. The Warburg-like effect has also been described as one of the changes that occur during metabolic reprogramming of immune cells [54].

In association with energy production, lipid biosynthesis is also an important metabolic change during the DC maturation process. In this regard, we found different proteins more abundant or exclusively stimulated by HP and associated with lipid biosynthesis (AGPAT3, HMGCS1, FDPS, CYP51A1, FADS2, ACSS2, PTPMT1, and FDXR). 

PTPMT1 is a lipid phosphatase which dephosphorylates phosphatidyl glycerophosphate to phosphatidylglycerol that may prevent intrinsic apoptosis, probably by regulating mitochondrial membrane integrity [55]. In PTPMT1 knockout hepatocellular carcinoma cells, mitochondria had altered morphology and generated less energy exhibiting slower growth, lower viability, and less aggressive phenotypes. These changes in mitochondrial function were accompanied by a reduction in cardiolipin levels, revealing that PTPMT enables cells to adapt to hypoxic conditions [56]. In this context, it has been described that DC functionality is affected by oxygen shortage conditions that are present in many of the microenvironments where DCs perform their functions [57]. Importantly, the conditional inactivation of PTPMT1 in human hematopoietic stem cells shows rapid inactivation of differentiation and cell divisions, which slows down the mitochondrial respiration and increases the anaerobic glycolysis [58]. 

CYP51A1 expression is increased in poor prognosis colorectal cancers in comparison with good prognostic tumors [59]. Remarkably, knockdown of CYP51A1 significantly inhibited the in vitro mobility and invasiveness of the lung cancer cell lines A549 and CL1-5 [60]. Moreover, overexpression of CYP51A1, HMGCS1, and FDFT1 caused an increase in migration/invasion capabilities of cells in vitro, suggesting a positive association between these proteins and invasiveness of lung cancer cells [60]. However, Araldi et al. have shown that, in macrophages, innate immune transcriptional downregulation of CYP51A1 induces lanosterol accumulation, promoting antimicrobial activity and favoring anti-inflammatory response [61]. Taken together, our results could be indicative of a role for HP in DCs metabolic reprogramming, which is relevant for achieving their complete mature phenotype and functionality [46,47].

We have previously demonstrated the role of specific DAMPs, such as HMGB1 and Calreticulin, in in vitro inducing a mature phenotype and enhancing antigen cross-presentation in human primary monocyte-derived DCs [18,22]. Here, proteins with previously described DAMP functions were also identified among the HP-induced proteome (Figure 2A, pink shadow), suggesting a role for HP in the induction of other DAMP proteins and its participation in a proinflammatory loop during DC activation. In this context, it has also been demonstrated that HP is associated with the activation of DCs in a murine model of skin transplantation [35]. Indeed, HP activates WT CD11c^+^ DCs in a MyD88-dependent manner, but TLR2 and TLR4-independent manner, which exhibited enhanced IL-6 and TNF-α production and priming of allogeneic T cells, compared with PBS-treated WT DCs and Myd88^–/–^ CD11c^+^ DCs [35]. 

During the maturation process, the phenotype of DCs undergoes several changes such as the upregulation of co-stimulatory molecules, the mayor histocompatibility complex (MHC) class I and II molecules, and the C-C chemokine receptor type 7 (CCR7) [8,11]. As mentioned above, we found several proteins associated with antigen presentation process, such as MHC class I (HLA-B and HLA-C) and class II molecules (HLA-DRB1, HLA-DRB5 and HLA-DQB1), among those exclusive or more abundant proteins in the HP-stimulated proteome. In addition, HP-stimulation was able to induce the expression of costimulatory molecules CD80 and CD86 as well as CD83, another well-known DC maturation marker, in primary immature DCs. Importantly, these molecules are considered relevant canonical markers of DC maturation [18,21,22,62,63]. 

Cytokine secretion is also relevant for a proper functionality of DC. Indeed, cytokines are considered an important signal during immunological synapse where the information transferred from DCs to naïve T cells defines their polarization [4,11,64]. HP-stimulated primary monocyte-derived DCs showed a higher secretion of cytokines IL-6, IL-8, and IL-12 when compared with unstimulated cells (Figure 3D). Interestingly, IL-6 and IL-12 have been associated with a DC phenotype for a proper Th1 immune response [62,65]. In this regard, and in a murine heterotopic cardiac transplant model, Shen et al. showed that HP modifies the intra-allograft milieu enhancing the levels of IL-6 but impairing the levels of the immunosuppressive IL-10 [36]. Additionally, we have previously showed that the melanoma-derived lysate TRIMEL induces the release of Th1-polarizing and pro-inflammatory cytokines such as IL-6, TNF-α, and IL-12 in therapeutically used monocyte-derived DCs [18,22].

One of the most relevant features of mature DCs is that they can migrate to draining secondary lymphoid organs where they can engage, in a proper cytokine context, antigen-specific naïve CD4^+^ and CD8^+^ T cells, triggering their activation, proliferation, and transference to peripheral tissues to perform effector functions [7,8]. Our proteomic data showed several proteins associated with changes in cell shape and cell migration upon HP-stimulation (Figure 2A, dark yellow shadow). Moreover, CCR7 expression was higher in HP-stimulated cells when compared with unstimulated cells. These results are also in line with our result showing that HP enhances in vitro transmigration of monocyte derived DCs in response to CCL19. Of note, CCR7 is recognized as a critical receptor for DC migration into draining lymph nodes [6,66], a process that is facilitated by the chemokines CCL19 and CCL21 that are expressed by the stromal cells close to the T cell area of lymph nodes [67,68,69,70]. The importance of CCR7 in the DCs migration and T cell activation has been demonstrated in CCR7-deficient knockout (KO) mice, where lymph nodes are almost completely depleted of naïve T cells and DCs and, by contrast, the T cell population is augmented in blood, spleen, and bone marrow [71,72]. Moreover, these KO mice have also altered antibody levels and diminished delayed hypersensitivity (DTH) responses [71,72]. In the same context, CCL19 KO mice showed that this chemokine is essential for DC homing to lymph nodes [68,73], and in corresponding transgenic mice, the ectopic expression of CCL19 resulted in the retention of DCs in these tissues and prevention of their migration to secondary lymphoid organs [74].

Mature DC phenotype, capable of leading to either Th1, Th2, Th17, or regulatory T cells (Tregs) among other adaptive immunity profiles, is triggered by different stimuli that lead to qualitatively different maturation states, suggesting that, depending on the nature of the stimulus, DCs interpret signals from the microenvironment through PRRs, for example TLRs [75]. Our data showed that in vitro stimulation of primary human monocyte-derives DCs with HP was able to: (i) induce exclusive and more abundant specific proteins that are associated with different mature DCs-associated characteristics; (ii) induce the expression of the canonical mature DC-associated markers CD80, CD83, and MHC-I; (iii) enhance the secretion of the pro-inflammatory cytokines IL-6, IL-8, and IL-12; and (iv) stimulate the transmigration in response to CCL19. In this context, we can speculate that the mature DC-associated phenotype induced by the lysate TRIMEL on ex vivo generated human primary DCs for therapeutic purposes [18,21,22] can be, at least in part, due to the HP present in the lysate. 

These results add relevant information about the impact of HP, a self-generated molecule, in the DC maturation process. This knowledge could help to develop new ex vivo manipulation procedures of DCs for therapeutical purposes, not only for the treatment of cancer but also autoimmune and neurodegenerative diseases.

## 4. Materials and Methods

### 4.1. Patients and Healthy Donors

Peripheral blood mononuclear cells (PBMC) were obtained by a leukapheresis procedure from three patients with advanced (stage IV) malignant melanoma previously treated with a reported TAPCells vaccination protocol [18,21]. Additionally, PBMC from four healthy donors, from the Blood Bank Service, Clinical Hospital, University of Chile, were obtained. The present study was performed in agreement with the Helsinki Declaration and approved by the Bioethical Committee for Human Research of the Clinical Hospital, University of Chile. All patients and healthy donors signed an informed consent form.

### 4.2. Cell Lines and the Melanoma Cell Lysate TRIMEL

Human melanoma cell lines (Mel1, Mel2 and Mel3), established from three tumor infiltrated lymph nodes from patients with metastatic HLA-A2+ stage IV melanoma and positive for several melanoma associated antigens, were cultured in RPMI-1640 medium (Invitrogen, Waltham, MA, USA) containing 10% FBS (Invitrogen, Waltham, MA, USA) until 90–95% confluence. Before use, all the cell lines were tested by PCR techniques, to check the absence of potentially infecting virus or mycoplasma. The presence of contaminating bacteria was also ruled out by periodical culture testing in agar. The allogeneic cell lysate TRIMEL was prepared as previously described [18,21]. Briefly, the three different melanoma cell lines were mixed in equal proportions (1 × 10^7^ cells for each cell line), resuspended in the therapeutic AIM–V medium (GIBCO, Carlsbad, CA, USA) at a concentration of 4 × 10^6^ cells/mL, HS-treated by incubating the cells one hour at 42 °C, then two hours at 37 °C and finally lysed by performing three freeze-thaw cycles using liquid nitrogen. The obtained cell lysates were sonicated, snap frozen, and stored at −80 °C, until further use [5]. Three independently produced batches of the lysate TRIMEL were prepared.

### 4.3. Ex Vivo Generation of Human Primary Monocyte Derived DCs

Human DCs were ex vivo generated from adherent monocytes isolated from PBMC of healthy donors and patients with stage IV malignant melanoma. PBMC were cultured in serum-free therapeutic AIM-V medium (GIBCO, Carlsbad, CA, USA) at a concentration of 13 × 10^6^ cells/mL in six well plates (BD Biosciences, Hershey, PA, USA) at 37 °C and 5% CO_2_ for 2 h. Thereafter, non-adherent cells were removed, and the adherents (monocytes) were maintained and incubated for 22 additional hours in the presence of 500 U/mL recombinant human IL-4 (rhIL-4) and 800 U/mL of rhGM-CSF (US Biological, Swampscott, MA, USA) in order to induce DC-lineage differentiation as previously described [18]. The obtained cytokine-activated monocytes (AM), which showed an immature DC-like phenotype, were then stimulated for 24 additional hours with 10 ng/mL of HP (Abcam, Cambridge, UK), the lysate TRIMEL (100 µg/mL) as positive control or 10 ng/mL of insulin (INS; Novus Biologicals, Centennial, CO, USA) as negative protein control. In addition, all the in vitro experiments were performed using unstimulated iDCs (AM) as internal control.

### 4.4. Cell Lysis and Protein Extraction–Digestion

A cell pellet containing 4 × 10^6^ cells was resuspended in 1 mL of lysis solution (0.2%ProteaseMax/10% acetonitrile (ACN)/50 mM ammonium bicarbonate (AmBic)). Cell lysis was performed over 10 min with the aid of rigorous vortexing. The lysate was kept at 95 °C for 5 min and then subjected to 15 min sonication (30% amplitude, 3:3 pulse) with a Branson sonicator. Samples were centrifuged at 14,000 rpm over 7 min at room temperature, and the precipitate was discarded. The total concentration of proteins was determined using a bicinchoninic acid assay (Pierce BCA assay kit, Thermo Fisher Scientific Inc., Waltham, MA, USA). Proteins were reduced by adding DTT to a final concentration of 10 mM and incubated for 30 min at 50 °C, and then alkylated via incubation with iodoacetamide for 30 min at room temperature. Proteins (80 µg) were digested by adding 2 µg of trypsin (Sequencing Grade Modified Trypsin, Promega, Madison, WI, USA) and incubated at 37 °C for 9 h. The digest was rigorously vortexed over 5 min. Digestion was terminated by the addition of 5% acetic acid. Samples were cleaned and desalted using C18 StageTips (Thermo Fisher Scientific Inc., Waltham, MA, USA), dried using a SpeedVac and resuspended in water with 0.1% formic acid.

### 4.5. Mass Spectrometry (MS)

Peptide mixture was injected into an Ultimate 3000 nanoflow LC system (Thermo Fisher Scientific Inc., Waltham, MA, USA) in-line coupled to a QExactive mass spectrometer (Thermo Fisher Scientific Inc., Waltham, MA, USA). The chromatographic separation of the peptides was achieved using a 25 cm long in-house packed column (C18-AQ ReproSil-Pur^®^, Dr. Maisch GmbH, Tübingen, Germany) at 55 °C with the following gradient: 4–30% ACN in 89 min, 26–95% ACN for 5 min and 95% ACN for 8 min all at a flow rate of 250 nL/min.

The MS acquisition method comprised one survey full scan ranging from *m*/*z* 300 to *m*/*z* 1650 acquired with a resolution of R = 140,000 at *m*/*z* 200 and AGC target value of 5 × 10^6^, followed by data-dependent higher-energy collisional dissociation fragmentation scans from maximum 16 most intense precursor ions with a charge state ≥2. For dependent scans, the following parameters were used: precursor isolation width 4 Da, AGC target value of 2*105, and normalized collision energy 26. Scans were acquired in profile mode with a resolution of R = 17,500.

### 4.6. Protein Identification and Quantification

The MS raw data were analyzed with the MaxQuant software (version 1.5.3.30, Max Planck Institute of Biochemistry, Munich, Germany). A false discovery rate (FDR) of 0.01 for proteins and peptides and a minimum peptide length of six amino acids were required. The mass accuracy of the precursor ions was improved by the time-dependent recalibration algorithm of MaxQuant. The Andromeda search engine was used to search the MS/MS spectra against the Uniprot human database (containing 90,482 entries) combined with 262 common contaminants and concatenated with the reversed versions of all sequences. Enzyme specificity was set to trypsin. Further modifications were cysteine carbamidomethylation (fixed) as well as protein N-terminal acetylation, asparagine and glutamine deamidation, and methionine oxidation (variable). A maximum of two missed cleavages was allowed. Peptide identification was based on a search with an initial mass deviation of the precursor ion of up to 7 ppm. The fragment mass tolerance was set to 20 ppm on the *m*/*z* scale. Only proteins quantified with at least two peptides were considered for quantitation.

### 4.7. Flow Cytometry Analysis

The cells were phenotypically characterized by flow cytometry using the following conjugated antibodies (Abs): mouse anti human-HLA-ABC-FITC, CD80-FITC, CD83-FITC, CD86-FITC, CCR7-FITC, and CD11c-PE-Cy7 (eBioscience, San Diego, CA, USA). Briefly, cells were gently removed from the culture plates using cell scrapers. Then, the cells were centrifuged at 1000 rpm for 5 min at 4 °C, washed with PBS and incubated with Abs for 30 min. After being washed twice with PBS, samples were acquired on a FACSVerse (BD Biosciences, Hershey, PA, USA) and analyzed using FlowJo software (Tree Star, Inc., Ashland, OR, USA). Cell viability was verified through the viability dye LIVE/DEAD (Thermo Fisher Scientific Inc., Waltham, MA, USA). All the analyses were made in the CD11c+ cell population of each condition and sample.

### 4.8. Cytokine Production Analysis

The secretion of cytokines by unstimulated iDCs (AM) and HP-stimulated cells was analyzed by flow cytometry, using a Cytometric Bead Array (CBA; BD Biosciences, Hershey, PA, USA) kit and according to the manufacturer’s recommendations. To this, the concentrations of IL-12, TNF-α, IL-10, IL-6 and IL-8 were analyzed in supernatants obtained from 24 h co-cultures of AM or HP-stimulated cells with CD-40L expressing fibroblast (1:1 ratio). The samples were acquired in a FACSVerse cytometer (BD Biosciences, Hershey, PA, USA).

### 4.9. In Vitro Transmigration Assay

Cell migration of AM, TRIMEL, INS, and HP-stimulated cells in response to CCL19 (10 ng/mL) was evaluated using a 48-well Transwell double chamber (Neuroprobe, Gaithersburg, MD, USA). Briefly, the lower chambers were loaded with media containing 30 μL per well of 10 ng/mL of CCL19 or AIM-V (negative control). Thereafter, corresponding inserts (upper chamber) with 8 µm pore size polycarbonate filters previously incubated for 24 h in fibronectin 1 mg/mL (Invitrogen, Carlsbad, CA, USA) were placed into the respective lower chamber. Finally, each well of the upper chamber was loaded with 50 μL of the cell suspension at a concentration 1 × 10^6^ cells/mL. After four hours of incubation at 37 °C, the upper inserts with the polycarbonate filter were removed. The polycarbonate filter was fixed in 100% methanol, stained with hematoxylin, washed twice with PBS, and placed onto a glass slide where the cells on the upper surface of the filter were mechanically removed. This last step allowed us to maintain the cells that were able to pass through the filter’s pore to the opposite side. Dry filters were further analyzed using an inverted microscope model DM IL (Leica Microsystems, Wetzlar, Germany). Cells considered to have migrated through the pores were counted from five representative fields for each well in at least three independent experiments. Results were expressed as a migration index, which is the ratio between the number of migrated cells towards CCL19 and the number of migrated cells towards the medium alone for each cell condition studied.

### 4.10. Data Analysis

Data were obtained from at least three independent experiments. One-way ANOVA or Kruskal–Wallis test for nonparametric variables were used to compare the significance of differences among groups. Paired two-tailed Student’s *t*-test or Mann–Whitney test were used to compare differences between groups. Data were expressed as mean ± standard deviation (SD) and statistically analyzed using Prism 6 software (GraphPad, San Diego, CA, USA). Differences were considered significant at *p* < 0.05. Analysis of the data provided by MaxQuant was performed in the R scripting and statistical environment. Differences in relative protein abundances between samples were assessed by a moderated *t*-test using a limma package [76]. Benjamini–Hochberg correction for multiple comparisons was used. Gene set enrichment analysis and visualization of protein–protein interaction networks were performed using STRING software (http://string-db.org/. Accessed date 25 October 2021) [77].

## 5. Conclusions

Taken together, our data support that HP induces a specific proteomic profile and a mature-associated phenotype on ex vivo generated primary human monocyte-derived DCs. These results add relevant information about the impact of HP, a self-generated molecule, in a DC maturation process and that, in this context, HP could be considered as a DAMP molecule.

This knowledge could help to develop new ex vivo manipulation procedures of DCs for therapeutical purposes, not only for the treatment of cancer but also autoimmune and neurodegenerative diseases.

## Figures and Tables

**Table 1:** Currently known functions of the 83 selected gene-proteins regulated by HP (showed in Figure 1C). The list of proteins was previously filtered by exclusivity or abundance (log2(Fold Change)) > 1, when comparing HP vs. AM.

Gene	ID (Uniprot)	Full Name (Uniprot)	Protein Subgroup	Function (Gene Ontology)
TAP2	A0A0G2JLV0	Antigen peptide transporter 2	E	i. Antigen processing and presentation of endogenous peptide antigen via MHC class I ii. Transmembrane transport
FDXR	A0A0A0MTN9	NADPH:adrenodoxin oxidoreductase, mitochondrial	E	NADPH-adrenodoxin reductase activity
NDUFA6	R4GN43	NADH dehydrogenase [ubiquinone] 1 alpha subcomplex subunit 6	E	i. NADH dehydrogenase (ubiquinone) activity
TJP2	A0A1B0GTW1	Tight junction protein ZO-2	E	i. Cell adhesion molecule binding ii. Guanylate kinase activity
SAE1	M0QX65	SUMO-activating enzyme subunit 1	E	Regulation of actin cytoskeleton organization
SLAMF7	B4DW98	SLAM family member 7	E	Identical protein binding
BAIAP2	B4DWA1	Brain-specific angiogenesis inhibitor 1-associated protein 2	E	Regulation of actin cytoskeleton and membrane organization
SLC25A20	C9JPE1	Mitochondrial carnitine/acylcarnitine carrier protein	E	Acyl carnitine transmembrane transporter activity
KRT14	CON_P02533	Keratin, type I cytoskeletal 14	E	i. Keratin filament binding, ii. Structural constituent of cytoskeleton
BZW2	Q75MG1	Basic leucine zipper and W2 domain-containing protein 2	E	Cadherin binding
ACADS	E9PE82	Short-chain specific acyl-CoA dehydrogenase, mitochondrial	E	i. Acyl-CoA; butiryl-CoA dehydrogenase activity, ii. Flavin adenine dinucleotide binding
CRYAB	E9PR44	Alpha-crystallin B chain	E	i. Amyloid-beta; identical protein; metal ion; microtubule; and unfolded protein binding ii. Structural molecule activity
CD1C	H0Y6Y6	T-cell surface glycoprotein CD1c	E	i. Endogenous; exogenous lipid antigen binding, ii. Glycolipid binding
CLUH	I3L2B0	Clustered mitochondria protein homolog	E	mRNA binding
SLC16A3	O15427	Monocarboxylate transporter 4	E	Lactate and monocarboxylic transmembrane acid transport
AK1	Q5T9B7	Adenylate kinase isoenzyme 1	E	Adenylate kinase activity and ATP binding
SLC25A4	P12235	ADP/ATP translocase 1	E	i. ATP:ADP antiporter activity ii. proton transmembrane transporter activity
ACADSB	P45954-2	Short/branched chain specific acyl-CoA dehydrogenase	E	i. Short-branched-chain-and acyl-CoA dehydrogenase activity ii. Identical protein binding
RECQL	P46063	ATP-dependent DNA helicase Q1	E	DNA unwinding involved in DNA replication and repair
LIG3	P49916-4	DNA ligase 3	E	i. ATP; DNA; zinc ion binding,
MFAP1	P55081	Microfibrillar-associated protein 1	E	RNA binding
TUBB2A	Q13885	Tubulin beta-2A chain	E	Microtubule cytoskeleton organization
MORC3	Q14149	MORC family CW-type zinc finger protein 3	E	Innate immune response, protein stabilization and maintenance of protein location in nucleus
SMEK1	Q6IN85-2	Serine/threonine-protein phosphatase 4 regulatory subunit 3A	E	Regulatory subunit of SMEK1
ATL2	Q8NHH9-5	Atlastin-2	E	i. GTP; identical protein binding ii. GTPase activity
DCP1A	Q9NPI6-2	mRNA-decapping enzyme 1A	E	i. mRNA; kinesin and identical protein binding ii. Enzyme activator and hydrolase activity
ACSS2	Q9NR19	Acetyl-coenzyme A synthetase, cytoplasmic	E	i. Lipid biosynthetic process ii. AMP and ATP binding
CHMP5	Q9NZZ3	Charged multivesicular body protein 5	E	Vacuolar transport, lysosome organization and negative regulation of cell death
ATXN10	Q9UBB4-2	Ataxin-10	E	Cilium assembly and nervous system development
WDR37	Q9Y2I8	WD repeat-containing protein 37	E	N/A
COA3	Q9Y2R0	Cytochrome c oxidase assembly factor 3 homolog	E	Mitochondrial cytochrome c oxidase assembly
HLA-DRB1	Q9GIY3	HLA class II histocompatibility antigen, DRB1-14 beta chain	MA	MHC class II receptor activity
CCDC58	C9JQ41	Coiled-coil domain-containing protein 58	MA	N/A
HLA-DQB1	A2AAZ0	HLA class II histocompatibility antigen, DQ beta 1 chain	MA	MHC class II receptor activity
CCL3	P10147	C-C motif chemokine 3	MA	Chemoattractant and protein kinase activity
HLA-B	P30493	HLA class I histocompatibility antigen, B-55 alpha chain	MA	Peptide antigen and signaling receptor binding
SLC39A7	Q92504	Zinc transporter SLC39A7	MA	Zinc ion transmembrane transporter activity
FADS2	O95864	Fatty acid desaturase 2	MA	i. Oxidoreductase activity ii. Protein binding
FBP2	O00757	Fructose-1,6-bisphosphatase isozyme 2	MA	i. Catalytic and hydrolase activity ii. Metal ion binding
HLA-DRB5	Q30154	HLA class II histocompatibility antigen, DR beta 5 chain	MA	MHC class II protein complex binding
ITGAE	P38570	Integrin alpha-E	MA	Integrin and metal ion binding
PCCB	F8WBI9	Propionyl-CoA carboxylase beta chain, mitochondrial	MA	Ligase activity
DHX38	Q92620	Pre-mRNA-splicing factor ATP-dependent RNA helicase PRP16	MA	i. RNA and nucleic acid binding ii. Helicase and ATP hydrolisis activity
HMGCS1	Q01581	Hydroxymethylglutaryl-CoA synthase	MA	Protein homodimerization activity
PTPMT1	Q8WUK0-2	Phosphatidylglycerophosphatase and protein-tyrosine phosphatase 1	MA	Protein tyrosine/serine/threonine phosphatase activity
HLA-C	A2AEA2	HLA class I histocompatibility antigen, Cw-7 alpha chain	MA	Peptide antigen binding
HLA-B	P30685	HLA class I histocompatibility antigen, B-35 alpha chain	MA	Peptide antigen and signaling receptor binding
RNF17	Q9BXT8-4	RING finger protein 17	MA	Metal ion and protein binding
GNPDA1	P46926	Glucosamine-6-phosphate isomerase 1	MA	Hydrolase and deaminase activity
HLA-B	Q04826	HLA class I histocompatibility antigen, B-40 alpha chain	MA	Peptide antigen and signaling receptor binding
DEFA3	P59666	Neutrophil defensin 3	MA	Protein homodimerization activity
PPCS	Q9HAB8-2	Phosphopantothenate--cysteine ligase	MA	Protein homodimerization activity
DCD	P81605	Dermcidin; Survival-promoting peptide; DCD-1	MA	Peptidase activity
ATP5E	Q5VTU8	ATP synthase subunit epsilon, mitochondrial	MA	Proton-transporting ATP synthase activity
DHRS4	Q9BTZ2-8	Dehydrogenase/reductase SDR family member 4	MA	Carbonyl reductase (NADPH) activity
HDDC3	H0YNP9	Guanosine-3,5-bis(diphosphate) 3-pyrophosphohydrolase MESH1	MA	Metal ion binding
SETD3	Q86TU7	Histone-lysine N-methyltransferase setd3	MA	Transcription coactivator and histone methyltransferase activity
POLE3	Q9NRF9	DNA polymerase epsilon subunit 3	MA	Chromatin DNA binding
FDPS	P14324-2	Farnesyl pyrophosphate synthase	MA	i. Transtransferase activity ii. Metal ion and RNA binding
UBE2G2	P60604	Ubiquitin-conjugating enzyme E2 G2	MA	Ubiquitin conjugating enzyme activity
KRT1	P04264	Keratin, type II cytoskeletal 1	MA	i. Structural constituent of skin epidermis ii. Protein heterodimerization activity
HLA-DRB1	Q29974	HLA class II histocompatibility antigen, DRB1-16 beta chain	MA	MHC class II protein complex binding
RDH11	Q8TC12-3	Retinol dehydrogenase 11	MA	Cellular detoxification of aldehyde
CHMP2A	O43633	Charged multivesicular body protein 2a	MA	Protein binding
AZGP1	P25311	Zinc-alpha-2-glycoprotein	MA	Protein transmembrane transporter activity
AGPAT3	Q9NRZ7-2	1-acyl-sn-glycerol-3-phosphate acyltransferase gamma	MA	Phospholipid biosynthetic process
DAD1	F5GXX5	Dolichyl-diphosphooligosaccharide-protein glycosyltransferase subunit DAD1	MA	Enzyme activator activity
C11orf54	E9PR95	Ester hydrolase C11orf54	MA	Metal ion binding
CD1A	P06126	T-cell surface glycoprotein CD1a	MA	Protein and lipid antigen binding
KRT10	CON_P13645	Keratin, type I cytoskeletal 10	MA	i. Structural constituent of skin epidermis ii. Protein heterodimerization activity
CYP51A1	A0A0C4DFL7	Lanosterol 14-alpha demethylase	MA	i. Heme and iron ion binding ii. Cholesterol biosynthetic process
LSM5	B8ZZF8	U6 snRNA-associated Sm-like protein LSm5	MA	mRNA splicing
DTX3L	Q8TDB6	E3 ubiquitin-protein ligase DTX3L	MA	i. Ubiquitin protein ligase activity ii. Histone binding
GABARAP	H6UMI1	Gamma-aminobutyric acid receptor-associated protein	MA	i. Autophagosome assembly ii. Microtubule cytoskeleton organization
BAG5	Q9UL15	BAG family molecular chaperone regulator 5	MA	Protein kinase and chaperone binding
ABCD3	P28288-2	ATP-binding cassette sub-family D member 3	MA	Protein binding
NUTF2	P61970	Nuclear transport factor 2	MA	i. Small GTPase binding ii. Structural constituent of nuclear pore
RBM10	A0A0A0MR66	RNA-binding protein 10	MA	RNA; miRNA; metal ion and protein binding
TIMM8A	O60220	Mitochondrial import inner membrane translocase subunit Tim8A	MA	Metal ion and protein binding
SAMSN1	Q9NSI8	SAM domain-containing protein SAMSN-1	MA	Phosphotyrosine residue and RNA binding
PTRHD1	Q6GMV3	Putative peptidyl-tRNA hydrolase PTRHD1	MA	Hydrolase activity
KRT9	P35527	Keratin, type I cytoskeletal 9	MA	Structural constituent of cytoskeleton
HSPA6	P17066	Heat shock 70 kDa protein 6	MA	i. Stress response ii. ATP, enzyme, misfolded protein, unfolded protein, heat shock protein and ubiquitin protein ligase binding iii. Protein folding chaperone

E, exclusive proteins (n = 31); MA, more abundant proteins (n = 52).

## Data Availability

Not applicable.

## Supplementary Materials

The following supporting information can be downloaded at: https://www.mdpi.com/article/10.3390/ijms23136882/s1.
