# Supplementary material for: Haptoglobin Induces a Specific Proteomic Profile and a Mature-Associated Phenotype on Primary Human Monocyte-Derived Dendritic Cells
Source: Int J Mol Sci. 2022 Jun 21;23(13):6882. doi: 10.3390/ijms23136882 (PMC9266681; doi:10.3390/ijms23136882)
Supplement: Supplementary file 1 [file ijms-23-06882-s001.zip › ijms-1759429-supplementary.pdf]

Supplementary Table S1. Exclusive and shared proteins among study groups.

	HP	TRIMEL	AM
**HP**	TAP2, BAIAP2, SLC25A20, BZW2, SLC16A3, RECQL, SMEK1, ATL2, WDR37	FDXR, NDUFA6, TJP2, SAE1, SLAMF7, KRT14, ACADS, CRYAB, CD1C, CLUH, AK1, SLC25A4, ACADSB, LIG3, MFAP1, TUBB2A, MORC3, DCP1A, ACSS2, CHMP5, ATXN10, COA3	FBP2, RNF17, POLE3, AZGP1, CYP51A1, TSPO, ALOX5, MRPL19, TGFBI, ARFGAP1, PRF1 NUP155, MYO9B, NDUFB6, APPL1, AP2S1, BRI3BP, TOMM34, AHCTF1, TMED5, NMI, RASGRP2, ZPR1, GOLGA3, EPN1, SGSH, WDR36, ACSL4, SCAF4, CC2D1A, FAM107B, SERPINB6, SCYL1, NDRG2, FAM96B, PFDN4, HIP1, JMJD6, RBM47, ARHGAP9, CASP8, PRTN3, DR1, ELMO2, PTPN18, SKIV2L, TMEM205, PLXND1, WASH1, GTF2F1, TRADD, EMG1, VPS51, PPP4R1, EMC8, MMAB, MGST1, PURB, METAP1, DCK, ZFYVE16, UTRN, IRF2BP2, RNF181
**TRIMEL**	FDXR, NDUFA6, TJP2, SAE1, SLAMF7, KRT14, ACADS, CRYAB, CD1C, CLUH, AK1, SLC25A4, ACADSB, LIG3, MFAP1, TUBB2A, MORC3, DCP1A, ACSS2, CHMP5, ATXN10, COA3	MYEF2, PMEL, NEDD4, COPS3	MYH10, MYO1D, CKB, CRMP1, AKR1B15, STXBP1, PMPCA, SEC24A, TRIP10, ERLEC1, MSH2, TXLNA, FAM134C, VPS11, LARP4, PPIH, NKIRAS2, ITGB5, NDUFAF2, ABR NUP98, OAS3, NUDT16, UBXN6, RABGAP1, SULT1A4
**AM**	FBP2, RNF17, POLE3, AZGP1, CYP51A1, TSPO, ALOX5, MRPL19, TGFBI, ARFGAP1, PRF1 NUP155, MYO9B, NDUFB6, APPL1, AP2S1, BRI3BP, TOMM34, AHCTF1, TMED5, NMI, RASGRP2, ZPR1, GOLGA3, EPN1, SGSH, WDR36, ACSL4, SCAF4, CC2D1A, FAM107B, SERPINB6, SCYL1, NDRG2, FAM96B, PFDN4, HIP1, JMJD6, RBM47, ARHGAP9, CASP8, PRTN3, DR1, ELMO2, PTPN18, SKIV2L, TMEM205, PLXND1, WASH1, GTF2F1, TRADD, EMG1, VPS51, PPP4R1, EMC8, MMAB, MGST1, PURB, METAP1, DCK, ZFYVE16, UTRN, IRF2BP2, RNF181	MYH10, MYO1D, CKB, CRMP1, AKR1B15, STXBP1, PMPCA, SEC24A, TRIP10, ERLEC1, MSH2, TXLNA, FAM134C, VPS11, LARP4, PPIH, NKIRAS2, ITGB5, NDUFAF2, ABR NUP98, OAS3, NUDT16,	KIAA1033, SEC61A1, MITF, TMED2, MAPRE2, CECR, PEF1

Supplementary Table S2. Proteomic profile of selected exclusive and more abundant proteins in HP group compared with AM- and TRIMEL- groups.

Proteins	TRIMEL vs. AM	HP vs. AM	TRIMEL vs. HP
TAP2	--	A in AM	A in TRIMEL
FDXR	A in AM	A in AM	5.79
NDUFA6	A in AM	A in AM	0.91
TJP2	A in AM	A in AM	1.98
SAE1	A in AM	A in AM	1.51
SLAMF7	A in AM	A in AM	2.30
BAIAP2	--	A in AM	A in TRIMEL
SLC25A20	--	A in AM	A in TRIMEL
KRT14	A in AM	A in AM	0.92
BZW2	--	A in AM	A in TRIMEL
ACADS	A in AM	A in AM	2.26
CRYAB	A in AM	A in AM	4.15
CD1C	A in AM	A in AM	1.08
CLUH	A in AM	A in AM	3.03
SLC16A3	--	A in AM	A in TRIMEL
AK1	A in AM	A in AM	3.21
SLC25A4	--	A in AM	12.13
ACADSB	A in AM	A in AM	3.40
RECQL	--	A in AM	A in TRIMEL
LIG3	A in AM	A in AM	3.92
MFAP1	A in AM	A in AM	0.90
TUBB2A	A in AM	A in AM	1.73
MORC3	A in AM	A in AM	4.68
SMEK1	--	A in AM	A in TRIMEL
ATL2	--	A in AM	A in TRIMEL
DCP1A	A in AM	A in AM	4.93
ACSS2	A in AM	A in AM	1.42
CHMP5	A in AM	A in AM	0.76
ATXN10	A in AM	A in AM	1.19
WDR37	--	A in AM	A in TRIMEL
COA3	A in AM	A in AM	2.27
HLA-DRB1	27.93	10.60	2.63
CCDC58	1.07	9.91	0.11
L-DQB1	2.71	9.03	0.30
CCL3	1.21	8.40	0.14
HLA-B	A in TRIMEL	4.83	A in TRIMEL
SLC39A7	8.14	4.12	1.98
FADS2	1.78	4.01	0.44
FBP2	A in TRIMEL	3.97	A in TRIMEL
HLA-DRB5	3.16	3.54	0.89

ITGAE	2.63	3.32	0.79
PCCB	1.08	3.28	0.33
DHX38	4.74	3.07	1.54
HMGCS1	1.62	3.00	0.54
PTPMT1	1.07	3.00	0.36
HLA-C	1.85	2.97	0.62
HLA-B	0.31	2.95	0.11
RNF17	A in TRIMEL	2.79	A in TRIMEL
GNPDA1	2.37	2.79	0.85
HLA-B	1.85	2.76	0.67
DEFA3; DEFA1	35.90	2.68	13.39
PPCS	1.98	2.58	0.77
DCD	2.05	2.55	0.80
ATP5E	1.61	2.53	0.64
DHRS4	1.04	2.49	0.42
HDDC3	1.11	2.46	0.45
SETD3	1.04	2.45	0.42
POLE3	A in TRIMEL	2.40	A in TRIMEL
FDPS	2.03	2.39	0.85
UBE2G2	0.65	2.36	0.28
KRT1	2.54	2.35	1.08
HLA-DRB1	1.00	2.33	0.43
RDH11	1.60	2.31	0.69
CHMP2A	1.35	2.25	0.60
AZGP1	A in TRIMEL	2.21	A in TRIMEL
AGPAT3	1.06	2.20	0.48
DAD1	2.39	2.18	1.09
C11orf54	1.78	2.18	0.82
CD1A	1.86	2.17	0.85
KRT10	1.71	2.12	0.80
CYP51A1	A in TRIMEL	2.12	A in TRIMEL
LSM5	1.51	2.11	0.72
DTX3L	1.25	2.11	0.59
GABARAP	2.47	2.08	1.19
BAG5	0.97	2.07	0.47
ABCD3	2.18	2.07	1.06
NUTF2	1.27	2.06	0.62
RBM10	3.96	2.05	1.93
TIMM8A	1.45	2.05	0.71
SAMSN1	1.79	2.03	0.88
PTRHD1	1.30	2.02	0.65
KRT9	2.52	2.01	1.25
HSPA6	1.39	2.00	0.69

*A in AM: Absent in AM group; A in TRIMEL: Absent in TRIMEL group.

Supplementary Table S3. List of top 20 over-expressed proteins in HP group compared with AM group. The abundance ratio for these proteins in the TRIMEL vs. AM comparison is also shown.

Protein name	Gene name	HP vs. AM	TRIMEL vs. AM
HLA class II histocompatibility antigen, DRB1-14 beta chain	HLA-DRB1	10.60	27.93
Coiled-coil domain-containing protein 58	CCDC58	9.91	1.07
HLA class II histocompatibility antigen, DQB1 beta chain	HLA-DQB1	9.03	2.71
C-C motif chemokine 3	CCL3	8.40	1.21
HLA class I histocompatibility antigen, B-55 alpha chain	HLA-B	4.83	A
Zinc transporter SLC39A7	SLC39A7	4.12	8.14
Fatty acid desaturase 2	FADS2	4.01	1.78
Fructose-1,6-bisphosphatase isozyme 2	FBP2	3.97	A
HLA class II histocompatibility antigen, DR beta 5 chain	HLA-DRB5	3.54	3.16
Integrin alpha-E	ITGAE	3.32	2.63
Propionyl-CoA carboxylase beta chain, mitochondrial	PCCB	3.28	1.08
Pre-mRNA-splicing factor ATP-dependent RNA helicase PRP16	DHX38	3.07	4.74
Hydroxymethylglutaryl-CoA synthase, cytoplasmic	HMGCS1	3.00	1.62
Phosphatidylglycerophosphatase and protein-tyrosine phosphatase 1	PTPMT1	3.00	1.07
HLA class I histocompatibility antigen, Cw-7 alpha chain	HLA-C	2.97	1.85
HLA class I histocompatibility antigen, B-35 alpha chain	HLA-B	2.95	0.31
RING finger protein 17	RNF17	2.79	A
Glucosamine-6-phosphate isomerase 1	GNPDA1	2.79	2.37
HLA class I histocompatibility antigen, B-40 alpha chain	HLA-B	2.76	1.85
Neutrophil defensin 3	DEFA3	2.68	35.90

*A: Absent in TRIMEL

Supplementary Table S4. Proteomic profile of exclusive and more abundant proteins in AM group compared with HP group.

AM	Proteins
**Exclusive proteins (n=33)**	KIAA1033, SEC61A1, MITF, TMED2, MAPRE2, CECR5, PEF1, MYH10, MYO1D, CKB, CRMP1, AKR1B15, STXBP1, PMPCA, SEC24A, TRIP10, ERLEC1, MSH2, TXLNA, FAM134C, VPS11, LARP4, PPIH, NKIRAS2, ITGB5, NDUFAF2, ABR, NUP98, OAS3, NUDT16, UBXN6, RABGAP1, SULT1A4
**More abundant proteins (n=31)**	HLA-DRB1, CALU, UBA3, ALDH1B1, METAP1, DCK, HDGFRP2, AP1G1, SUPT6H, HLA-DQA1, COX7C, STRN, ZFYVE16, UTRN, PDLIM4, APOH, TTR, NAA10, ARHGAP10, IRF2BP2, RNF181, PGLYRP2, HLA-C, CEP170, NELFB, GGCT, PDLIM2, EBNA1BP2, HMGA1, IDH3B, LPGAT1, CARD9, HLA-B

Supplementary Table S5. Proteomic profile of exclusive and more abundant proteins in TRIMEL group compared with AM group.

TRIMEL	Proteins
**Exclusive proteins (n=26)**	MYEF2, PMEL, NEDD4, COPS3, SLC25A4, AK1, FDXR, DCP1A, MORC3, CRYAB, LIG3, ACADSB, CLUH, SLAMF7, COA3, ACADS, TJP2, TUBB2A, SAE1, ACSS2, ATXN10, CD1C, KRT14, NDUFA6, MFAP1, CHMP5
**More abundant proteins (n=21)**	PGLYRP2, ABHD12, SERPINH1, ATAD1, PMPCA, MGLL, IPO5, GABARAP, DUSP3, RNASE3, KRAS, IPO9, ALDH18A1, SRC, AP1M1, FAM50A, GNB2L1, TUBA1A, ACOT7, GALE, TUBB4A
